# RUNX2, GPX3 and PTX3 gene expression profiling in cumulus cells are reflective oocyte/embryo competence and potentially reliable predictors of embryo developmental competence in PCOS patients

**DOI:** 10.1186/1477-7827-11-109

**Published:** 2013-11-26

**Authors:** Xin Huang, Cuifang Hao, Xiaofang Shen, Yuhua Zhang, Xiaoyan Liu

**Affiliations:** 1 Reproductive Medicine Centre, Affiliated Hospital of Qingdao Medical University, Yuhuangding Hospital of Yantai, 20 Yuhuangding Road East, Yantai, Shandong, China; 2 CapitalBio Corporation Center (Biochip National Engineering Research Center), Yantai Branch Center, 20 Yuhuangding Road East, Yantai, Shandong, China

**Keywords:** Embryo development, Fertilization, Gene expression, Human cumulus cells, Oocyte maturation, PCOS

## Abstract

**Background:**

Polycystic ovary syndrome (PCOS) is a common endocrine and metabolic disorder in women. The developmental competence of oocytes and embryos in PCOS patients is reduced to a certain extent (comparing to non-PCOS patients, the high quality embryo rate was decreased by 16% from the data of our centre) during the in vitro fertilization (IVF) process. Cross-talk between the oocyte and cumulus cells is critical for oocyte maturation and embryo competence. In this study, we have evaluated the transcription of specific genes in cumulus cells harvested from pre-ovulatory follicles of PCOS patients before IVF, according to individual oocyte nuclear maturity and developmental competence. Seven genes (*RUNX2*, *PSAT1*, *ADAMTS9*, *CXCL1*, *CXCL2*, *CXCL3*, and *ITGB5*) were targeted from our previous cDNA microarray data which isolated genes related to oocyte nuclear maturation in PCOS patients. Two additional genes which had been found to be associated with oocyte maturation or embryo quality in non-PCOS patients (*GPX3* and *PTX3*) were also studied.

**Methods:**

The mRNA expression levels of cumulus cells were detected by qRT- PCR.

**Results:**

Consistent with our previous cDNA microarray data, with the exception of *GPX3* and *PTX3*, the selected 7 genes were related to oocyte nuclear maturation in PCOS patients. Noticeably, the expression level of *RUNX*2 was lower in cumulus cells derived from oocytes that could develop into blastocysts than the level of expression from oocytes that could not. The *PTX3* expression level was significantly lower in cumulus cells from oocytes with two normal pronuclei than that from oocytes that formed >2 pronuclei (MPN) after fertilization. *GPX3* mRNA levels were decreased in cumulus cells isolated from oocytes that developed into blastocysts with high potential development competence.

**Conclusions:**

Several cumulus cell genes were associated with oocyte maturation, fertilization and embryo quality in PCOS patients. *RUNX2* and *GPX3* are candidate genetic markers in the monitoring of embryo quality for PCOS patients, whereas *PTX3* mainly played a role in fertilization process. Together with morphological evaluation, cumulus cells genes may serve as biomarkers of oocyte and embryo selection during the IVF process for PCOS patients and may advance our understanding of PCOS.

## Background

Polycystic ovary syndrome (PCOS) is a common endocrine and metabolic disorder in women [1-3] which is characterized by increased circulating androgen levels, anovulatory infertility, and, frequently, insulin resistance and hyperinsulinemia. During in vitro fertilization (IVF), although sufficient oocytes are usually retrieved from PCOS patients who are under controlled ovarian stimulation (COS), high-quality mature oocytes are limited in number [4-7]. The paucity of high-quality mature oocytes may result from endocrine and intra-ovarian paracrine interactions in PCOS patients which could change the microenvironment for oocyte development during the folliculogenesis and reduce the developmental competence of oocytes [8,9]. Follicular growth and maturation are prerequisites to oocyte fertilization and subsequent early embryo development [10-12]. In antral follicles, granulosa cells line the follicle wall, whereas cumulus cells encircle the oocyte to form the cumulus-oocyte-complex (COC). Cross-talk between cumulus cells (CCs) and oocytes in the follicle is understood to be a pivotal process in oocyte maturation and metabolism [13,14]. The impact of the oocyte-cumulus cell relationships on both maturation and fertilization ability have been well-established [15,16]. Previous researchers have reported that gene expressions of CCs could be used as a tool to predict oocyte competence or embryo development [14,17-24].

In our previous report [25], the expression levels of 59 genes exhibited significant differences during different phases of nuclear maturation (MII and MI) in CCs of PCOS patients. The results may indicate that the expression of these CCs genes are associated with oocyte maturation in PCOS patients. Moreover, several genes (*LHCGR*, *TNIK* and *SOCS3*) may serve as biomarkers of embryo competence in CCs of PCOS patients. To evaluate the roles of other genes in the CCs of PCOS patients during developmental processes, including oocyte maturation, fertilization and embryo development, we analysed a panel of 9 genes. Seven of these genes, [Runt-related transcription factor 2 (*RUNX2*), Phosphoserine aminotransferase 1 (*PSAT1*), ADAM metallopeptidase with thrombospondin type 1 motif 9 (*ADAMTS9*), chemokine (C-X-C motif) ligand 1,2,3 (*CXCL1*, *CXCL2*, *CXCL3*), and integrin β5(*ITGB5*)] were chosen based on differences in their expression at different oocyte nuclear maturity stages (MII or MI) in cumulus cells of PCOS patients [25]. Another two genes [glutathione peroxidase 3 (*GPX3*) and pentraxin 3 (*PTX3*)] were selected because they have previously been related to oocyte maturation or embryo quality [11,18,22].

The aim of this study was to evaluate the expression of these genes in cumulus cells from PCOS patient during different developmental stages (oocyte maturation, fertilization and blastocyst formation). The results would help us identify whether the developmental competence of the oocytes/ embryos of PCOS patients are related to the specific expression patterns of these genes and whether these genes are clinically important and could offer a new potential strategy for selecting competent oocytes or embryos in PCOS patients.

## Methods

### Patients and IVF treatment

This project was approved by the Institutional Ethical Review Board of Affiliated Hospital of Qingdao Medical University (Yuhuangding Hospital of Yantai). PCOS patients referred to our center for in vitro fertilization (IVF) were included in this study after written informed consent. All of the PCOS patients were characterized by the presence of two or more of the following features: chronic oligo-ovulation or anovulation, androgen excess and polycystic ovaries. We excluded patients with Cushing’s syndrome, congenital adrenal hyperplasia, and androgen-secreting tumors. The inclusion criteria of the recruited PCOS patients in this study were as follows: age ranging between 30 and 38 years, BMI ranging between 20 and 26 kg/m^2^, number of obtained oocytes ranging between 13 and 19 per cycle, number of obtained blastocysts ranging between 4 and 9 per cycle, basal serum LH/FSH more than 2.0, serum androgen more than 0.5 ng/ml, and normal spermiogram of the partner according to the WHO criteria. Ovarian stimulation and oocyte retrieval protocols were performed as our previously described in all PCOS patients [25].

### Retrieval of cumulus cells

The method of retrieving cumulus cells and the process of oocyte insemination and culture were performed as described previously [25]. In brief, after COC retrieval, a proportion of the CCs surrounding a single oocyte were removed using a sharp needle, lysed in 80 ml of RLT buffer (RNeasy Mini Kit, Qiagen) supplemented with 1% (v/v) 2-β-mercaptoethanol (M-3148; Sigma, Lyon, France), and stored at −80°C (CCs from one oocyte per vial) until RNA extraction. Oocytes were further inseminated and embryos were cultured in sequential media of SAGE (CooperSurgical, Leisegang Medical, Berlin) individually in 20 ul droplets covered by mineral oil. Embryos were transferred or vitrified on Day 3 and the other embryos were cultured to blastula stage on Day 5–6.

### Assessment of oocyte and embryo quality

The morphological characteristics of the oocytes and embryos were individually recorded. Oocytes were denudated to assess the maturation stage 3 h after insemination. The oocytes were first classified into two categories based on nuclear status: (i) Immature MI oocytes exhibiting no polar bodies (PB) or immature oocytes at the germinal vesicle stage (GV), and (ii) mature MII oocytes that extruded a clearly visible PB. Fertilization was observed at 16–18 h after insemination and zygotes were classified into two groups: (i) oocyte exhibiting normal fertilization (2 pronuclei, 2PN) and (ii) oocyte exhibiting abnormal fertilization (more than 2 pronuclei, MPN). Oocytes with no pronuclei (0PN) were discarded. On Day 2 (44–46 h post-IVF), individually cultured embryos were evaluated according to the number of blastomeres, the fragmentation rate and the presence of multinucleated blastomeres. Embryos with one or several multinucleated blastomeres and un-cleaved embryos were excluded. On Day 3, embryos containing at least 7 cells, <10% fragmentation and no multinucleated blastomeres were classified as ‘top quality embryo (TQE)’. Others were classified as ‘weak or low grade embryo (WLGE)’ [24]. Two or three embryos were transplanted into the uterus of patients on Day 3, whereas the others were frozen or cultured to the blastocyst stage. The blastocysts were scored on the expansion of the blastocoel cavity, the cohesiveness of the inner cell mass, and trophectodermal cells [26]. The best blastocyst quality on Day 5, i.e. blastocyst score 1 = BL 3–5, AA or AB (inner cell mass/ trophectoderm , ICM/TE) with ≤10% fragmentation. The good blastocyst quality on Day 5, blastocyst score 1 or 2 with score 2 = BL 1,2 or BL 3–5 with BA or BB (ICM/TE score) with ≤20% fragmentation.

### Experimental design and the groups of cumulus cells

**Figure 1:**
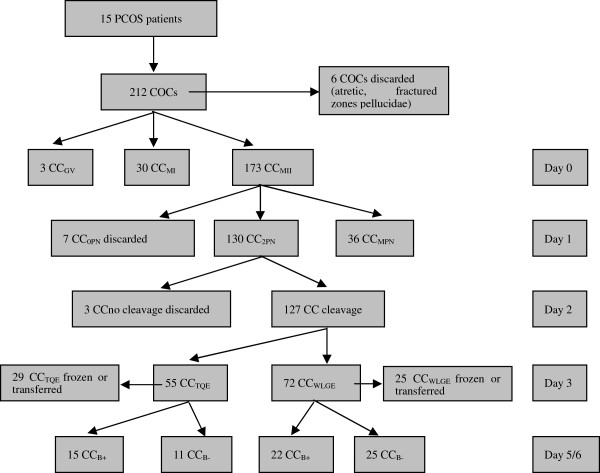
**Distribution tree of COCs involved in the study.** A total of 212 COCs were retrieved from the 15 PCOS patients. Except for 6 COCs which are atretic or with fractured zones pellucidae were discarded, there have 3 GV stage COCs, 30 MI stage COCs, and 173 MII stage COCs. On Day 1 (16–18 hours after insemination), 130 CCs separated from oocytes with normal fertilization (2PN) and 36 CCs separated from mature oocytes with abnormal fertilization (MPN) were classed into a “CC_2PN_” and “CC_MPN_” group, respectively. On Day 3, 54 of 127 embryos that developed from oocytes with normal fertilization (2PN) were transplanted or frozen, and their corresponding CCs were classified into six subgroups randomly. The other 73 embryos were cultured to the blastocyst stage. Cumulus cells from oocytes yielding blastulas after 5/6 days in vitro culture were classified as the “CC_B+_” group (n = 37), and cumulus cells from oocytes that stopped developing at the embryo stage after Day 6 were divided into “CC_B-_” groups (n = 36). COC, cumulus-oocyte-complex; CC_GV_, cumulus cells from GV oocyte; CC_MI_, cumulus cells from MI oocyte; CC_MII_, cumulus cells from MII oocyte; CC_0PN_, cumulus cells from oocyte which formed no pronuclei (0PN) after fertilization; CC_2PN_, cumulus cells from oocyte which formed two pronuclei (2PN) after fertilization; CC_MPN_, cumulus cells from oocyte which formed multi pronuclei (MPN) after fertilization; CC_WLGE_, cumulus cells from oocyte yielding a weak or low grade embryo at Day3; CC_TQE_, cumulus cells from oocyte yielding a top quality embryo at Day 3; CC_B-_, cumulus cells from oocyte which stopped development at the embryo stage at Day 5/6; CC_B+_, cumulus cells from oocyte yielding a blastocyst after 5/6 days of in vitro culture.

According to different stages of oocytes or embryos, the corresponding cumulus cells were divided into different groups, viz.: CC_MI/GV_, CC_MII_, CC_2PN_, CC_MPN_, CC_B+_, CC_B-_. Each group had ≥3 replicates. Each subgroup, containing 9–12 cumulus cells, represented a biological replicate. The distribution of the collected COCs is depicted in Figure 1. In our experiment, a total of 212 COCs were retrieved from the 15 PCOS patients. Among these COCs, only 3 GV stage COCs were retrieved and were combined into the CC_MI/GV_ groups (n = 33). In each CC_MI/GV_ subgroup, there were ten CC_MI_ and one CC_GV_. On Day 1 (16–18 hours after insemination), 130 CCs separated from oocytes with normal fertilization (2PN) and 36 CCs separated from mature oocytes with abnormal fertilization (MPN) were classed into a “CC_2PN_” and “CC_MPN_” group, respectively. On Day 3, 54 of 127 embryos that developed from oocytes with normal fertilization (2PN) were transplanted or frozen and their corresponding CCs were classified into 6 subgroups of CC_2PN_ while the other 73 embryos were cultured to the blastocyst stage. In each subgroup, there are 9 cumulus cells randomly from 3 or 4 PCOS patients. Cumulus cells from oocytes yielding blastulas (top or good blastocyst quality)after 5–6 days in culture were classified as the “CC_B+_” group (n = 37), and cumulus cells from oocytes that arrested at the cleavage stage after Day 6 were divided into “CC_B-_” groups (n = 36). The number of CCs and biological replicates in the different groups are shown in Table 1.

**Table 1:** The number of CCs and biological replicates in different groups

**CC groups**	**Number of CCs**	**Biological replicates**
*CC*_*MI/GV*_	33	3
*CC*_*MII*_	173	15
*CC*_*2PN*_	130	12
*CC*_*MPN*_	36	3
*CC*_*B+*_	37	3
*CC*_*B-*_	36	3

### RNA extraction and reverse transcription

The cumulus cells of each subgroup were pooled together to extract total RNA and were analysed individually. Total RNA isolation was performed using the Qiagen RNeasy Mini Kit (Qiagen, Hilden, Germany) according to the manufacturer’s instructions. This RNA isolation kit significantly reduces contamination from both genomic DNA and proteins. The RNA quality was assessed using an Agilent 2100 Bioanalyzer (Agilent Technologies, Kista, Sweden). Only samples with RNA integrity number (RIN) >6.0 in the cumulus cells subgroups were included. A good correlation between RIN values >5.5 and the outcome of a real-time PCR experiment has been shown and RIN values ≥ 5.5 are considered as high quality [27]. The first-strand complementary synthesis reaction was performed using the PrimeScript RT reagent Kit (Perfect Real time; TaKaRa Biotechnology, Dalian, China).

### qRT-PCR

qRT-PCR was performed on 9 genes (*RUNX2*, *PSAT1*, *ADAMTS9*, *CXCL1*, *CXCL2*, *CXCL3*, *ITGB5*, *GPX3* and *PTX3*). Primers for all the target genes were designed using the Universal Probe Library software (Roche Diagnostics, Roche Applied Science) and were chosen to be intron spanning. Amplification reactions were conducted using SYBR Premix Ex Taq (Perfect Real Time) (TaKaRa Biotechnology, Dalian, China) and an ABI PRISM 7300 system. The gene-specific qRT-PCR primers used are listed in Table 2. To normalize the expression level, the housekeeping gene *GAPDH* was used because its expression was stable in all CCs groups. The PCR thermal cycling conditions were 95°C for 10 min for polymerase activation and the initial denaturation step, followed by 40 cycles with denaturation at 95°C for 15 s, annealing at 60°C for 60 s and extension at 72°C for 30 s. A melting curve analysis was recorded at the end of the amplification to evaluate the absence of contaminants or primer dimers. Each set of qRT-PCR reactions was repeated three times, and the fold change in the expression of each gene was analysed using the DDCt method [28].

**Table 2:** The primer sequences (5’-3’) used in qRT-PCR

**Gene symbol**	**Accession nr**	**Forward primer**	**Reversed primer**	**Fragment length**
*GAPDH*	NM_002046	TGTTGCCATCAATGACCCCTT	CTCCACGACGTACTCAGCG	202
*RUNX2*	NM_001015051.3	TCCAGGAGGACAGCAAGAAGT	TGTCACTGTGCTGAAGAGGCT	182
*PSAT1*	NM_021154 .3	ATGGGCTTGGTTCTGGAGTG	GGCCAGCTTCTGAACGTCTT	324
*ADAMTS9*	NM_182920.1	AGGGTCGTTTTAGCATCAACC	TCCACAGTAACCACCGCATT	149
*CXCL1*	NM_001511.2	CCCAAACCGAAGTCATAGCC	CCTTCAGGAACAGCCACCAG	158
*CXCL2*	NM_002089.3	CCCAAACCGAAGTCATAGCC	CAGGAACAGCCACCAATAAGC	154
*CXCL3*	NM_002090.2	CACTCAAGAATGGGAAGAAAGC	GCAGGAAGTGTCAATGATACGC	142
*ITGB5*	NM_002213.3	GACCGGAGGGAGTTTGCAA	CCTCGGAGAAGGAAACATCAGT	170
*GPX3*	NM_002084.3	GGCTTTGTCCCTAATTTCCAG	AAAGTTCCAGCGGATGTCGT	171
*PTX3*	NM_002852.3	GCATCTCCTTGCGATTCTGTT	CATTCCGAGTGCTCCTGACC	165

### Statistical analysis

Student’s t-test of independent data was used for statistical analysis (SPSS Inc., Chicago, IL). The differences among groups were considered significant when the p-value was <0.05.

## Results

### Transcripts levels of the target genes according to oocyte nuclear maturity in PCOS patients

**Figure 2:**
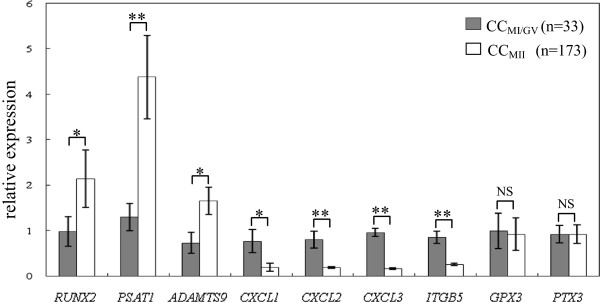
**Transcripts levels of the target genes (*RUNX2*, *PSAT1*, *ADAMTS9*, *CXCL1*, *CXCL2*, *CXCL3*, *ITGB5*, *GPX3 *and *PTX3*) according to oocyte nuclear maturity in PCOS patients.** mRNA expression of candidate genes (*RUNX2*, *PSAT1*, *ADAMTS9*, *CXCL1*, *CXCL2*, *CXCL3*, *ITGB5*, *GPX3* and *PTX3*) in cumulus cells of PCOS patients, organized according to oocyte nuclear maturity stage (MI/GV stage versus MII stage). The signal intensity for each gene is shown on the y-axis in arbitrary units determined by qRT-PCR analysis with *GAPDH* as an endogeneous reference. * indicates a significant difference in gene expression between CC categories (** p < 0.01, * p < 0.05). The results are presented as the means ± SEM. CC_MI/GV_: cumulus cells from oocyte at the MI stage or GV stage; CC_MII_: cumulus cells from oocyte at the MII stage.

In the CC_MII_ (n = 173) compared to CC_MI/GV_ (n = 33) groups (Figure 2), mean transcript levels of three genes were significantly higher, with 2.18-, 3.37- and 2.26- fold increases observed for *RUNX2* (2.14 ± 0.63 versus 0.98 ± 0.33), *PSAT1* (4.38 ± 0.92 versus 1.3 ± 0.3), and *ADAMTS9* (1.65 ± 0.3 versus 0.73 ± 0.23), respectively, whereas the expression levels of four genes were significantly decreased in the CC_MII_ samples, with 4.05-, 4.21-, 6- and 3.27- fold decreases observed for *CXCL1* (0.19 ± 0.09 versus 0.77 ± 0.26), *CXCL2* (0.19 ± 0.02 versus 0.8 ± 0.18), *CXCL3* (0.16 ± 0.02 versus 0.96 ± 0.09) and *ITGB5* (0.26 ± 0.03 versus 0.85 ± 0.13), respectively. The transcripts levels of the other two genes (*GPX3* and *PTX3*) exhibited no significant differences between the CC_MII_ and CC_MI/GV_ groups.

### Transcripts levels of the target genes according to the fertilization process in PCOS patients

**Figure 3:**
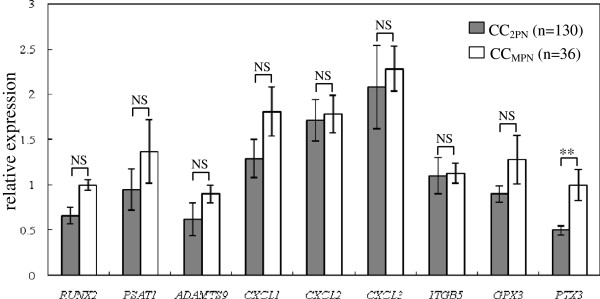
**Transcripts levels of the target genes (*RUNX2*, *PSAT1*, *ADAMTS9*, *CXCL1*, *CXCL2*, *CXCL3*, *ITGB5*, *GPX3 *and *PTX3*) according to the fertilization process in PCOS patients.** mRNA expression of candidate genes (*RUNX2*, *PSAT1*, *ADAMTS9*, *CXCL1*, *CXCL2*, *CXCL3*, *ITGB5*, *GPX3* and *PTX3*) in cumulus cells of PCOS patients, organized according to pronuclei formation after fertilization (normal two pronuclei versus multi pronuclei). The signal intensity for each gene is shown on the y-axis in arbitrary units determined by qRT-PCR analysis with *GAPDH* as an endogeneous reference. * indicates a significant difference in gene expression between CC categories (** p < 0.01, * p < 0.05). The results are presented as the means ± SEM. CC_2PN_: cumulus cells from oocyte which formed two normal pronuclei (2PN) after fertilization; CC_MPN_, cumulus cells from oocyte which formed multi pronuclei (MPN) after fertilization.

To evaluate whether target genes were involved in the fertilization process, the transcript levels of the target genes were detected in CC_2PN_ (n = 130) and CC_MPN_ (n = 36) groups (Figure 3). The *PTX3* expression level exhibits significant differences between CCs isolated from oocytes which formed two normal pronuclei (2PN) and that from oocytes which formed more than two pronuclei (MPN) after fertilization. The mean expression level of *PTX3* was 2- fold lower in the CC_2PN_ group than in the CC_MPN_ group (0.5 ± 0.05 versus 1 ± 0.17). The expression levels of the other 8 target genes exhibited no significantly changes.

### Transcripts levels of the target genes according to embryo development potential in PCOS patients

**Figure 4:**
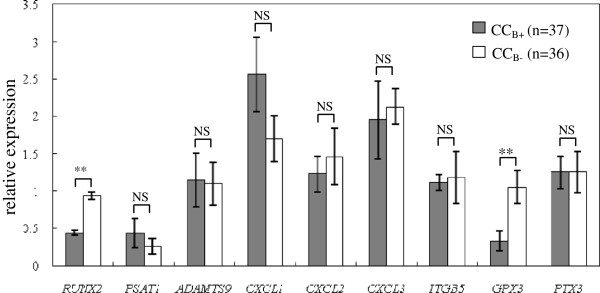
**Transcripts levels of the target genes (*RUNX2*, *PSAT1*, *ADAMTS9*, *CXCL1*, *CXCL2*, *CXCL3*, *ITGB5*, *GPX3 *and *PTX3*) according to embryo quality in PCOS patients.** mRNA expression of candidate genes (*RUNX2*, *PSAT1*, *ADAMTS9*, *CXCL1*, *CXCL2*, *CXCL3*, *ITGB5*, *GPX3* and *PTX3*) in cumulus cells of PCOS patients, organized according to oocyte development capability after normal fertilization. The signal intensity for each gene is shown on the y-axis in arbitrary units determined by qRT-PCR analysis with *GAPDH* as an endogeneous reference. * indicates a significant difference in gene expression between CC categories (** p < 0.01, * p < 0.05). The results were presented as the means ± SEM. CC_B+_: cumulus cells from oocyte yielding blastocyst after 5–6 days of in vitro culture; CC_B-_: cumulus cells from oocyte that had not developed into blastocyst at Day 5/6.

The transcripts levels of the target genes were then evaluated according to the outcome of the normal fertilized oocytes after 5–6 days of culture. Among the 9 target genes, two genes (*RUNX2* and *GPX3*) exhibited differential expression between the CC_B+_ (n = 37) and CC_B-_ (n = 36) groups (Figure 4), whereas the other 7 genes exhibited no significantly changes. The mean transcripts levels of *RUNX2* was decreased by 2.14- fold in the CC_B+_ group compared with the CC_B-_ group (0.44 ± 0.03 versus 0.94 ± 0.05), whereas the mean expression level of *GPX3* was decreased by 3.18- fold in the CC_B+_ group compared with the CC_B-_ group (0.33 ± 0.13 versus 1.05 ± 0.22).

## Discussion

PCOS is the most common heterogeneous endocrinopathy in women of reproductive age. Although patients suffering from PCOS benefit from assisted reproduction techniques (ARTs), oocyte competence and the pregnancy rates are compromised. Existing data demonstrates that oocyte competence in PCOS patients is altered [4-7,29,30]. Improving the competence of oocytes or embryos of PCOS patients will raise their probability of pregnancy and reduce the risk of multiple gestations. To date, non-invasive embryo selection has been based mainly on morphological features including the polar body (PB), zona pellucida, meiotic spindle, and cytoplasm. However, morphological evaluation is not a reliable predictor of oocyte competence and embryo quality [14]. Indeed, oocyte competence depends on the quality of the follicular microenvironment, and the presence of adequate bidirectional cumulus cell (CC)-oocyte signaling is critical for both oocyte and CC competence acquisition [10-12,14,20]. In this respect, the analysis of CCs, in conjunction with morphological criteria, is an appropriate approach for oocyte or embryo selection.

In our previous report [25], the expression levels of six genes which mainly associated with different signal pathways [two genes involved in the neuroactive ligand–receptor interaction pathway (*LHCGR* and *GRIN2A*), two genes involved in the Wnt signaling pathway (*SFRP4* and *TNIK*), one gene involved in the type 2 diabetes mellitus pathway (*SOCS3*), and one gene mediating angiogenesis (*ANGPTL1*)] were detected by qRT-PCR to evaluate whether they were related with oocyte/embryo quality. The results indicated that the molecular signatures (*LHCGR*, *TNIK*, and *SOCS3*) were associated with developmental potential from embryo to blastocyst stage and were proposed as biomarkers of embryo competence in PCOS patients. Now, in this study, the expression levels of additional seven genes which were mainly related with inflammatory response and development [inflammation-related genes (*CXCL1*, *CXCL2*, *CXCL3*), inflammation-related transcription factor (*RUNX2*), development (*ADAMTS9*), amino acid biosynthesis (*PSAT1*), cell-matrix adhesion (*ITGB5*)] from our cDNA microarray data and another two genes [glutathione peroxidase 3 (*GPX3*) and pentraxin 3 (*PTX3*)] which were proved to be related with oocyte maturation or embryo quality in previous reports were detected to investigate whether these genes were associated with embryo developmental potential in PCOS patients. This study is the second one in a row to evaluate the predictive value of CC gene expression for oocyte/embryo quality using qRT-PCR. Our hypothesis is that by this ‘cascade’ testing strategy, the strongest predictive genes which were associated with embryo quality in PCOS patients may be filtered out through the consecutive studies.

In the present research, the relative mRNA abundance of the selected 7 genes (*RUNX2*, *PSAT1*, *ADAMTS9*, *CXCL1*, *CXCL2*, *CXCL3*, *ITGB5*) in CCs isolated from oocytes at different nuclear maturity stages (MII and MI/GV) were consistent with our cDNA microarray data [25]. Compared to the list of DEGs (n = 25) which were related to oocyte nuclear maturity stage in non-PCOS patients [10], we confirmed that these selected 7 genes were all not identified in the Ouandaogo’s report. These data may indicate that there are different molecular mechanisms governing the process of oocyte nuclear maturation in PCOS and non-PCOS patients. For *GPX3* and *PTX3*, the expression levels of mRNA exhibited no significant differences between the CC-_MII_ and CC-_MI/GV_ groups in our research, and they were not included in the candidate genes isolated from cumulus cells according to oocyte nuclear maturity stages in non-PCOS patients [10]. These results suggested that *GPX3* and *PTX3* may play roles in biological process other than oocyte nuclear maturity. We therefore investigated whether the selected genes were involved in fertilization and embryo development.

### Genes were mainly associated with oocyte nuclear maturation in PCOS patients

Among the selected nine genes, the expression levels of six genes (*PSAT1*, *ADAMTS9*, *CXCL1*, *CXCL2*, *CXCL3*, *ITGB5*) exhibited significant differences in CCs from oocytes at different nuclear maturation stages. No change was observed between the CC-_2PN_ and CC-_MPN_ groups or between the CC-_B+_ and CC-_B-_ groups. The results implied that these genes may play roles during the oocyte nuclear maturation progress. In our previous report [25], the genes were identified as being involved in different biological processes as follows: amino acid biosynthesis (*PSAT1*), development (*ADAMTS9*), the inflammatory response (*CXCL1*, *CXCL2*, *CXCL3*) and cell-matrix adhesion (*ITGB5*).

The relative mRNA abundance of *PSAT1*and *ADAMTS9* in cumulus cells from oocytes at the MII stage was higher than that from oocytes at MI/GV stages. PSAT1, phosphoserine aminotransferase, is an enzyme implicated in serine biosynthesis and has been linked with cell proliferation in vitro [31]. PSAT1 plays an important role in the G2/M phase to modify the cell cycle and stimulate cell proliferation [32]. *ADAMTS9*, a gene involved in extracellular matrix binding, is widely expressed during mouse embryo development [33]. Furthermore, the extracellular matrix of CCs plays a major role in folliculogenesis and is crucial for ovulation, oviduct passage, and fertilization [34].

Comparing the CC-_MII_ group with the CC-_MI/GV_ group, the expression levels of four genes (*CXCL1*, *CXCL2*, *CXCL3*, *ITGB5*) were decreased. To our knowledge, these four genes, which are related with the inflammatory response (*CXCL1*, *CXCL2*, *CXCL3*) or cell–matrix adhesion (*ITGB5*), have been isolated from the CCs of PCOS patients for the first time by cDNA microarray in our previous research [25]. Though the role of chemokines during oocyte maturation is unclear, previous researches have demonstrated that the inflammatory response, which is induced by other chemokines (such as IL-8), increases the rates of meiotic arrest and the failure of germinal vesicle breakdown [35]. Indeed, chemokines have been implicated in a number of reproductive events, including ovulation, menstruation, embryo implantation, and parturition and disease conditions (i.e., endometriosis and cancer) [36,37]. *ITGB5* (integrin beta 5) has also been implicated in cell migration and angiogenesis during embryo implantation due to its apical expression on glandular and luminal epithelial cells [38]. Previous research has demonstrated that the expression of *ITGB5*, which regulated by chemokines (such as CX3CL1 and CCL14), could increase trophoblast adherence and migration at the maternal-fetal interface [39]. In the present research, we observed the same trend (down-regulation) in mRNA expression of four genes (*CXCL1*, *CXCL2*, *CXCL3*, *ITGB5*) during the oocyte maturation process. Although it remains to be clarified whether the expression of *ITGB5* is regulated by chemokines (CXCL1, CXCL2, CXCL3), our findings suggest that the expression of these genes in cumulus cells is negatively related to oocyte maturation and may be involved in embryo implantation.

### PTX3 may play an important role in the fertilization process in PCOS patients

*PTX3* is a downstream target gene of *GDF9*, which is a member of the transforming growth factor-β superfamily and plays an important role in promoting cumulus expansion [40]. Cumulus expansion is a critical process for normal oocyte development, ovulation, and fertilization [41,42]. PTX3 co-localises with hyaluronic acid throughout the cumulus matrix from the periphery to the zona pellucida and interacts with tumour necrosis factor α-induced protein 6 (TNFAIP6) instead of binding directly to hyaluronan (HA) [43]. Inactivation of *PTX3* by targeted mutagenesis has been reported to reduce the likelihood of fertilization, possibly through disruption of the structural integrity of the cumulus complex [44]. The relationship between *PTX3* levels and oocyte competence is, however, controversial. Only Zhang and colleagues have reported that *PTX3* mRNA levels exhibit significant differences between cumulus cells derived from unfertilized oocytes and from oocytes that developed to the eight-cell stage and led to the establishment of clinical pregnancies [18]. In previous studies, neither the *PTX3* transcript level [17,45] nor the PTX3 protein concentration in follicular fluid [46] exhibited significant association with oocyte quality. Our present results also show no significant difference between cumulus cells isolated from oocytes at different nuclear maturation stages (MII and MI stages) or cumulus cells derived from oocytes with different developmental competence (embryos that could develop to blastocyst stage or not). However, the *PTX3* expression level exhibited significant differences between cumulus cells isolated from mature oocytes that formed two normal pronuclei or multi- pronuclei after fertilization (CC-_2PN_ and CC-_MPN_ groups). So, we speculated that *PTX3* plays a key role in the fertilization process instead of oocyte maturation or embryo quality.

### RUNX2 and GPX3 may serve as biomarkers for embryo selection in the CCs of PCOS patients

RUNX2 is a transcription factor that has been shown to play a crucial role in cell differentiation. The expression of *RUNX2* was identified in rat ovary and was shown to be increased by the luteinizing hormone (LH) surge in preovulatory follicles and the luteal tissue [47]. Previous reports have confirmed the strong relationship between *RUNX2* expression and ovulation, luteinization and steroidogenesis [48]. Consistent with Papamentzelopoulou’s findings [49], our data also proved that *RUNX2* played an antagonistic role in regulating embryo development and could possibly be used as a genetic marker for projecting embryo quality. Recently, it was reported that the expression level of *RUNX2* was lower in the central stroma of PCOS ovaries than in those of non-PCOS ovaries. The results may have implications for the PCOS-related defects in the inflammation-like ovulatory process [50]. In our research, it is interesting that the transcript level of *RUNX2* to be higher in cumulus cells derived from oocytes at the MII stage than in those at the MI or GV stage of PCOS patients. Maybe the significant difference of *RUNX2* transcript levels between CC_MII_ and CC_MI/GV_ was also related with the defects of inflammatory response in immature oocytes.

The expression level of *GPX3* exhibited significant difference between the CC-_B+_ and CC-_B-_ groups. Consistent with previous researches which proved that *GPX3* was related with embryo development or pregnancy outcome [22,51], we observed that the expression level of *GPX3* was associated with embryo development and could be a projector of embryo quality in PCOS patients. *GPX3* is regulated by hypoxia through HIF-1 binding sites which detected only in glutathione peroxidase [52]. Hypoxia can induce the formation of reactive oxygen species (ROS) which can cause lipid peroxidation, enzyme inactivation and cell damage, thereby, leading to apoptosis [53]. This phenomenon occurs both in cumulus cells and in the oocyte [54]. Moreover, the hypoxia conditions and the higher concentration of ROS in follicular fluid are negatively associated with embryonic development and pregnancy outcomes [55,56]. Therefore, *GPX3* may indicate that hypoxic conditions could act as a negative regulator of embryo development in PCOS patients.

The strengths of this study were the well-diagnosed PCOS women according to the Rotterdam criteria and the cumulus cells were classified strictly according to the different development stages of oocyte/embryo. A limitation of the study was that the cumulus cells in each subgroup which derived from 3–4 PCOS patients were pooled together to extract RNA due to the total RNA purified from a single cumulus is so limited. This may cause an imbalance of the samples and limits the endpoints that can be analyzed. But the imbalance of the samples could not be avoided absolutely, unless all the cumulus cells were derived from the same patient. Moreover, because PCOS is a common heterogeneous endocrinopathy in women of reproductive age, the molecular roles of target genes in the development of oocyte/embryo need be further verified in the large-scale PCOS patients.

## Conclusions

In conclusion, our results support existing evidence that the expression profiling of cumulus cells may be project competence of oocytes and embryos. In our study, one gene, *RUNX2*, accurately predicted both oocyte maturity and embryo quality for PCOS patients. Two additional genes (*PTX3* and *GPX3*) were related to fertilization and embryo quality in PCOS patients, respectively. Cumulus cells, which are typically discarded during IVF, are therefore suitable for the determination of developmental potential of the oocyte and embryo. For PCOS patients, expression patterns of cumulus cells may provide an objective basis for embryo selection, and could further elucidate the complex molecular mechanisms governing oocyte and embryo development.

## Competing interests

The authors declare that they have no competing interests.

## Authors’ contributions

XH and CFH devised the study and participated in its design. XH and YHZ did the practical analysis, advised by CFH. XFS and XYL sampled the material and cultured the embyros. XH wrote the manuscript. XH, CFH and YHZ corrected the manuscript. All authors read and approved the final manuscript.
